# Hybrid Planning for Complex Brain Metastases in Linear Accelerator (LINAC)-Based Stereotactic Radiosurgery: A Dosimetric Comparison With Dynamic Conformal Arc (DCA) and Volumetric Modulated Arc Therapy (VMAT) Techniques

**DOI:** 10.7759/cureus.106356

**Published:** 2026-04-03

**Authors:** Hyunuk Jung, Jihyung Yoon, Yuwei Zhou, Michael T Milano, Kenneth Usuki, Dandan Zheng

**Affiliations:** 1 Radiation Oncology, University of Rochester, Rochester, USA

**Keywords:** brain metastases, dca, hybrid, hyperarc, linac-based srs, rapidarc, srs, vmat

## Abstract

Introduction: Dynamic conformal arc (DCA) and volumetric modulated arc therapy (VMAT) are widely used for linear accelerator-based single-isocenter multi-target stereotactic radiosurgery (SRS) in treating multiple brain metastases. DCA offers advantages in delivery efficiency, quality assurance, and dose gradient steepness. However, DCA produces suboptimal conformity for challenging targets, including large postoperative cavities, irregularly shaped lesions, or tumors adjacent to critical organs-at-risk (OARs). VMAT improves conformity for complex targets but may be unnecessary for simple lesions within the same treatment plan. BrainLAB, Munich, Germany, recently developed a hybrid planning technique that applies VMAT arcs to one challenging lesion while using DCA for the remaining targets within a single plan.

Methodology: Ten patients with 73 planning target volumes (PTVs) were retrospectively replanned using four techniques: DCA, Hybrid (BrainLAB Elements, v4.0), Varian RapidArc® (RA), and HyperArc (HA). Dosimetric endpoints included the Radiation Therapy Oncology Group (RTOG) conformity index (CI), Paddick conformity index (PCI), gradient index (GI), normal brain volume exposures (V23-V5 Gy), and maximum doses to critical organs at risk (OARs). Efficiency metrics included monitor units and beam-on time.

Results: All techniques achieved ≥95% PTV coverage. VMAT-based plans (RA, HA) demonstrated superior conformity (median CI 1.03-1.11, PCI 0.85-0.90) compared with DCA (CI 1.41, PCI 0.68). Hybrid planning (CI 1.40, PCI 0.71) improved overall conformity versus DCA and achieved VMAT-like performance for VMAT-applied targets (CI 1.07, PCI 0.89). DCA exhibited the lowest GI; Hybrid maintained the low GI for VMAT-applied targets, while improving conformity. HA minimized normal brain exposure, while RA produced highest V5-V8 Gy. Hybrid resembled DCA in high-dose volumes while avoiding RA’s extensive low-dose exposure. OAR maximum doses showed no significant differences. Hybrid had the longest beam-on time due to additional arcs.

Conclusions: Hybrid planning enhances conformity for challenging targets while preserving favorable DCA characteristics for remaining lesions. Despite longer beam-on times, Hybrid offers a practical solution for single-isocenter multi-target SRS cases involving irregular cavities or OAR-adjacent lesions.

## Introduction

The rapid development of stereotactic radiosurgery (SRS) and fractionated SRS (fSRS) has been propelled by advancements in treatment planning and delivery technologies [1]. Among these, linear accelerator (LINAC)-based single-isocenter multi-target SRS/fSRS has become widely used in clinical practice for treating multiple brain metastases [2,3]. Two primary planning strategies are commonly utilized in this context: dynamic conformal arc (DCA) and volumetric modulated arc therapy (VMAT) [4-9].

DCA offers a simpler, forward-planning approach that is well-suited for small, spherical brain metastases, and the scripted DCA technique (BrainLAB Elements Multiple Brain Mets (MME), BrainLAB, Munich, Germany) further increases its applicability for handling multiple targets and enhances inter-planner consistency. In contrast, VMAT uses inverse planning with higher modulation and is hence able to better conform the dose around complex target geometries. For single-isocenter multi-target SRS, the DCA approach has previously demonstrated advantages in QA efficiency, inter-planner consistency, delivery efficiency for a small number of targets, and superior gradient index when treating a larger number of targets [10,11]. However, DCA becomes less effective when treated targets include large postoperative cavities, highly irregular lesions, or targets near critical organs-at-risk (OARs), such as the brainstem. In these cases, DCA plans may show suboptimal conformity and insufficient target coverage to the challenging target, which can compromise overall plan quality.

To overcome these limitations, BrainLAB recently developed a hybrid planning technique in its Elements software. This method integrates both DCA and VMAT within a single plan: VMAT arcs are applied to one selected challenging lesion, while DCA is used for the remaining targets. By merging these complementary strategies, hybrid planning seeks to improve conformity and coverage for challenging lesions while maintaining the benefits of DCA for the rest of the targets.

This study aims to provide an initial dosimetric evaluation of the Elements Hybrid Planning Technique compared to traditional DCA (MME) and VMAT methods (RapidArc (RA) and HyperArc (HA)). We hypothesize that the Hybrid technique will effectively bridge the clinical gap by maintaining the steep dose gradients characteristic of DCA while achieving target conformity comparable to VMAT techniques for irregular or OAR-adjacent lesions.

## Materials and methods

Patient selection

A total of 10 patients with multiple brain metastases who had previously been treated at our institution were retrospectively chosen for this planning study. This study was approved by our institutional research study review board. The patient characteristics and treatment details are summarized in Table 1. The median age at treatment was 68.5 years (range: 59-82 years). In total, 73 planning target volumes (PTVs) were included, consisting of 22 targets in patients with postoperative resection cavities and 51 targets in patients with lesions located near or within the brainstem. The median PTV was 2.9 cc (range: 0.1-41.3 cc) for cavity-associated metastases and 0.5 cc (range: 0.1-10.9 cc) for brainstem-associated targets. Fractionation regimens followed standard clinical practice, with prescriptions ranging from 21 Gy in three fractions to 35 Gy in five fractions, depending on lesion size and location.

**Table 1:** Summary of patient characteristics and treatment parameters.

No.	Parameter	Value
1	Number of patients	10
	Multiple-mets with a cavity	5
	Multiple-mets with a target near/inside the brainstem	5
2	Median age	68.5
3	Total number of PTVs	73
	Multiple-mets with a cavity	22
	Range (#/patient)	2-6
	Multiple-mets with a target near/inside the brainstem	51
	Range (#/patient)	4-13
4	PTV volume (cc)	
	Multiple-mets with a cavity	
	Median	2.9
	Range	0.1-41.3
	Multiple-mets with a target near/inside the brainstem	
	Median	0.5
	Range	0.1-10.9
5	Prescribed dose	# of targets
	21 Gy in 3 fractions	4
	24 Gy in 3 fractions	8
	25 Gy in 5 fractions	1
	27 Gy in 3 fractions	57
	35 Gy in 5 fractions	3

Treatment planning

For each patient, four distinct SRS treatment plans were created: dynamic conformal arc (DCA) and Hybrid Planning (Hybrid) using BrainLAB Multiple Metastases Elements (version 4.0), and VMAT-based RA and HA using Varian Eclipse (Varian Medical Systems, Palo Alto, CA). All plans were developed using the same planning CT dataset, fused with high-resolution, contrast-enhanced T1-weighted MRI to ensure precise target and OAR delineation, and were delivered with 6 MV flattening filter-free (FFF) beams on the Varian Edge system equipped with high-definition MLC (Varian Medical Systems).

Target definition and prescription

The gross tumor volume (GTV) was defined as the enhancing lesion or postoperative cavity. The PTV was created by expanding the GTV with an additional 0.5 to 1.5 mm in all directions, in line with institutional standards for intracranial SRS. The margin size was chosen by the physician’s discretion, considering the size, location, and proximity of the lesion to critical structures. Plans were normalized such that at least 95% of the planning target volume (PTV) received the prescribed dose, while adhering to the recommendations of the American Association of Physicists in Medicine (AAPM) Task Group 101 (TG-101) and HyTEC, as well as institutional dose constraints for critical organs at risk, including the brainstem, optic nerves, and optic chiasm [12-14]. Additionally, a maximum dose of up to 150% of the prescription dose within the PTV was allowed. Treatment planning was conducted retrospectively by two experienced SRS planners following institutional protocols.

DCA and Hybrid planning technique

DCA plans were generated using a standardized single-isocenter, multi-target template that automatically optimizes couch angles, gantry rotation paths, and collimator rotations based on the target geometry. Each plan included five couch angles, and for each position, two partial arcs (clockwise and counterclockwise) were used, resulting in a total of ten arcs per plan. The arc paths were shaped to conform to individual PTVs, with automatic collimator angle selection to minimize MLC leakage and interleaf transmission.

The Hybrid technique was recently introduced in BrainLAB Elements version 4.0, which combines DCA and VMAT arcs within a single treatment plan. This workflow started with creating a standard DCA plan, as described earlier. Then, three additional partial VMAT arcs in three different existing couch angles were added to optimize the coverage for the selected challenging target. This approach aims to utilize the complementary benefits of both planning strategies within a unified plan.

All DCA and hybrid plans were calculated using the Monte Carlo algorithm with a 1 mm dose grid size.

RapidArc and HyperArc

Both RA and HA employed the VMAT technique. RA plans were created using a predefined template designed for Brain SRS/fSRS in Eclipse. The setup included four distinct couch angles (90°, 45°, 0°, and 315°), with two full or partial arcs (clockwise and counterclockwise) at each couch position. This resulted in up to eight arcs per plan. To ensure reproducibility and minimize inter-planner variability, the manual adjustment of collimator angles and optimization goals followed a standardized institutional protocol with fixed priority rankings for PTV coverage and OAR sparing. HA plans were generated using the automated stereotactic planning module. The template featured four non-coplanar arcs: one full 360° arc at couch 0°, and three half arcs at couch angles of 45°, 315°, and 270°, respectively. Collimator rotations were automatically optimized based on the geometric distribution of the PTVs during plan creation. HA’s automated workflow reduced manual input while maintaining high conformity and steep dose fall-off. For both RA and HA plans, the dose was calculated using the AcurosXB algorithm (version 15.6.06) with a 1 mm dose grid size.

Dosimetric evaluation

Plan quality was evaluated using standard SRS dosimetric indices, as outlined in our previous publication [11]. The RTOG conformity index (CI), Paddick conformity index (PCI), and gradient index (GI) were calculated for each PTV. CI is defined as CI = TV/PTV, where TV is the treated volume enclosed by the 100% prescription isodose, and PTV is the PTV. PCI is defined as PCI = (TV_PIV_)^2^/(TV*PIV), where TV_PIV_ is the target volume covered by the prescription isodose, TV is the target volume, and PIV is the prescription isodose volume. GI is defined as GI = PIV_half_/PIV, where PIV_half_ is the prescription isodose volume as half of the prescription isodose, and PIV is the prescription isodose volume. Target coverage was defined as the percentage of PTV receiving ≥100% of the prescription dose. We also assessed the volume of normal brain, which excludes GTV volumes from the total brain volume, receiving doses of ≥23, 18, 12, 8, and 5 Gy, respectively. Additionally, the maximum dose (minimum dose to the hottest 0.035cc of the structure) to the brainstem, chiasm, and optic nerves was measured. Finally, the total beam-on time for each planning technique was recorded to compare delivery efficiency.

Statistical analysis

A Wilcoxon signed-rank test was used to statistically assess the extracted dosimetric parameters across the four techniques. The difference was considered statistically significant if *P* < 0.05.

## Results

**Figure 1:**
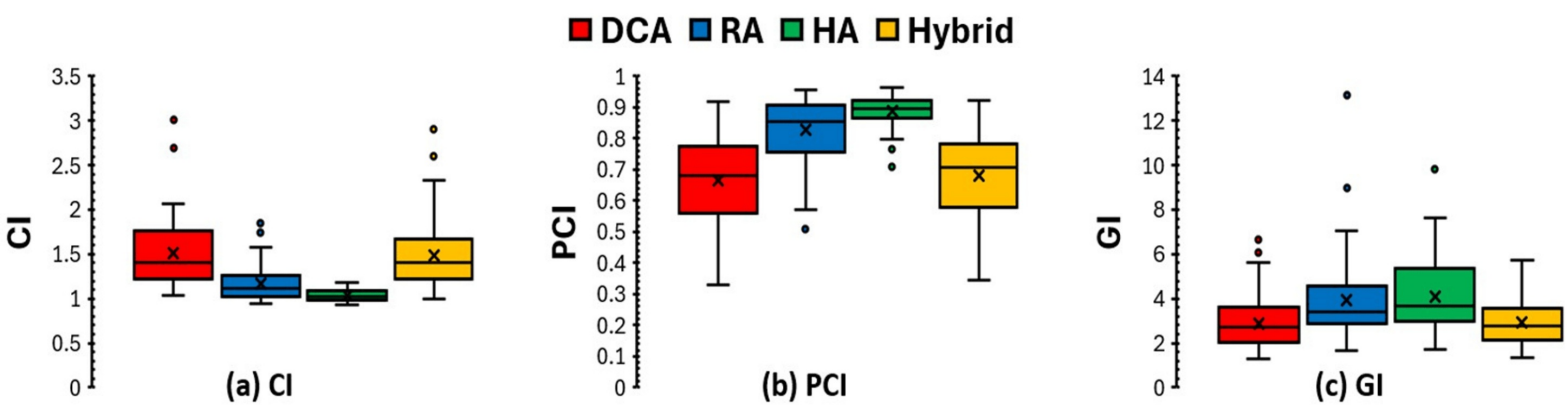
Comparison of the conformity index (CI), Paddick conformity index (PCI), and gradient index (GI) for total PTVs comparing the four different planning techniques. PTV, planning target volume; DCA, dynamic conformal arc; RA, RapidArc; HA, HyperArc

**Figure 2:**
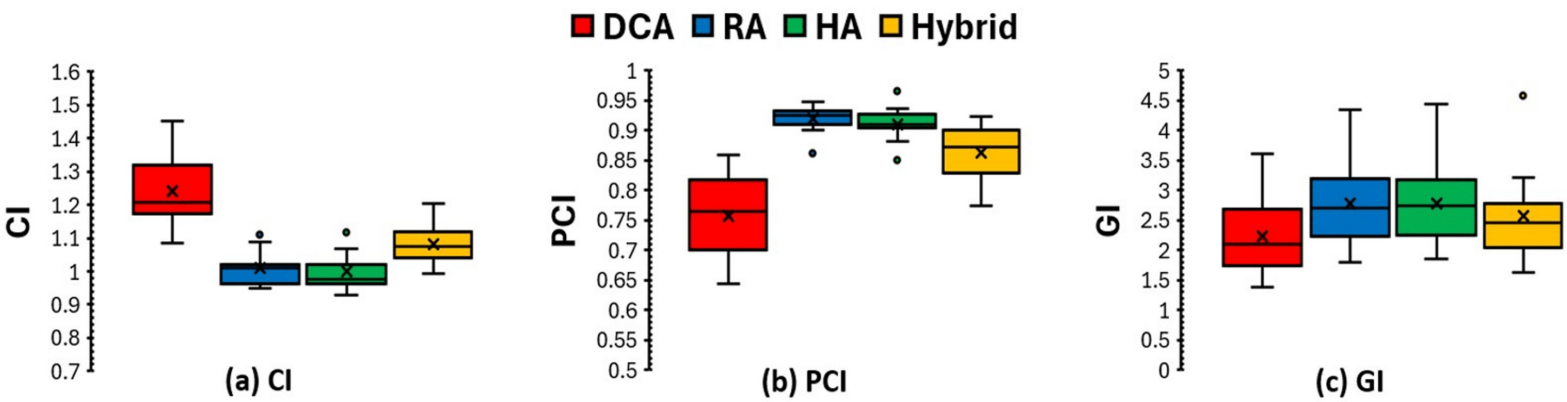
Comparison of the CI, PCI, and GI for VMAT-applied PTVs comparing the four different planning techniques. CI, conformity index; PCI, Paddick conformity index; GI, gradient index; VMAT, volumetric modulated arc therapy; PTV, planning target volume; DCA, dynamic conformal arc; RA, RapidArc; HA, HyperArc

All four planning techniques achieved clinically acceptable PTV coverage, with at least 95% of the PTV receiving the prescribed dose. Figure 1 shows a comparison of the CI, PCI, and GI across targets in all plans using four different planning techniques. The analysis indicates that HA and RA plans, which employ the VMAT technique, demonstrate superior CI and PCI compared to DCA and Hybrid plans. The median CI and PCI for HA plans were 1.03 and 0.90, respectively, which were comparable to those for RA plans (1.11 and 0.85) and superior to those for DCA (1.41 and 0.68) and hybrid plans (1.40 and 0.71) (*P* < 0.05 for all comparisons). When focusing only on the subset of targets where VMAT arcs were used within the Hybrid plans, the CI and PCI were similar to those achieved with RA and HA plans (Figures 2a-2b).

**Figure 3:**
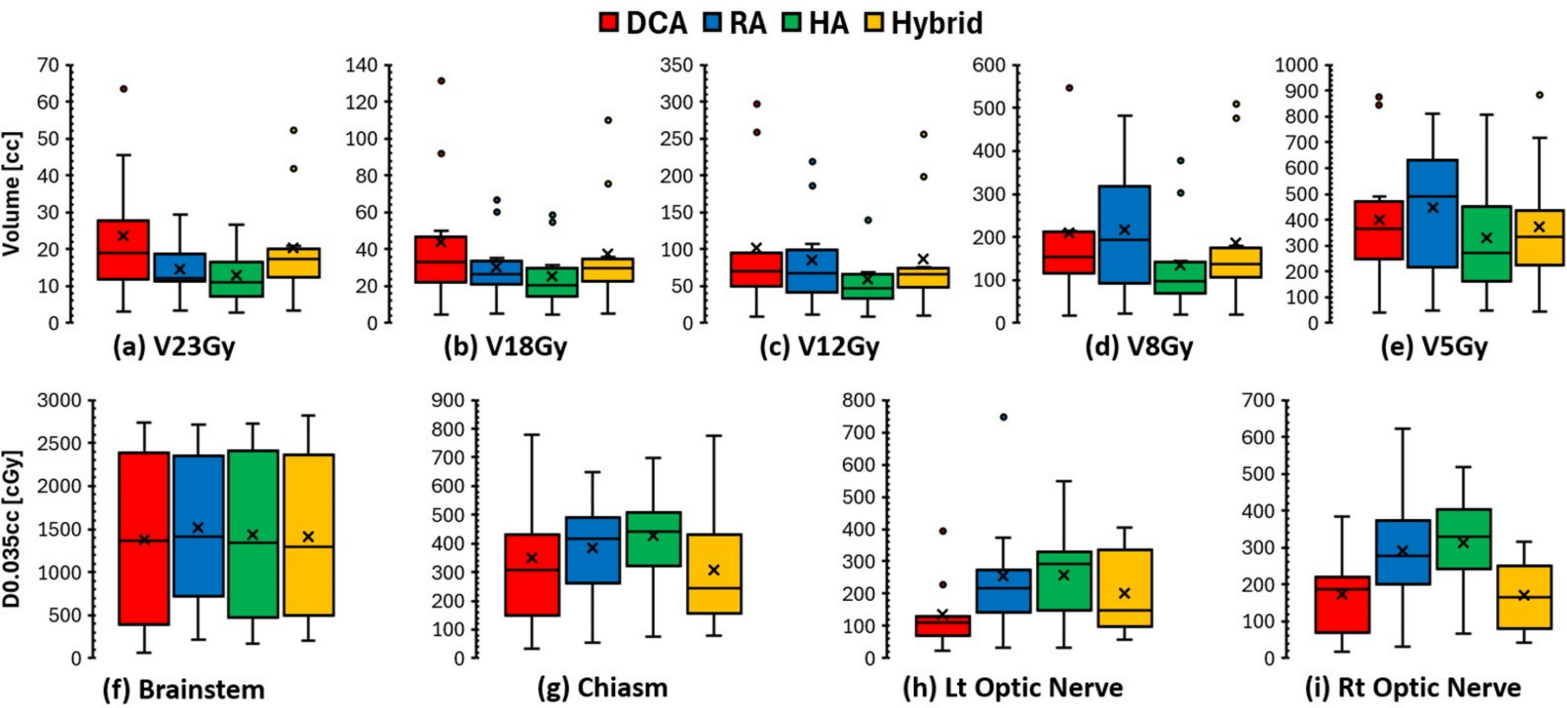
Comparison of the normal brain volume receiving (a) 23 Gy, (b) 18 Gy, (c) 12 Gy, (d) 8 Gy, and (e) 5 Gy, and the maximum dose to the (f) brainstem, (g) chiasm, (h) left optic nerve, and (i) right optic nerve for the four planning techniques. DCA, dynamic conformal arc; RA, RapidArc; HA, HyperArc

The GI was lowest for DCA plans (2.70 ± 1.12), reflecting their steep dose fall-off due to less beam modulation. Hybrid planning maintained GI values (2.79 ± 1.02) similar to DCA while improving conformity, especially for VMAT applied targets (Figure 2), resulting in a good balance between conformity and gradient. VMAT-based plans (RA and HA) showed slightly higher GI values (3.39 ±1.87 for RA and 3.65 ± 1.64 for HA), linked to broader low-dose spread, especially in RA plans, as shown in Figure 3.

Figures 3a-3e show box plots comparing the volume of normal brain tissue receiving ≥ 23, 18, 12, 8, and 5 Gy. Overall, the HA plans achieved the lowest doses to normal brain tissue across all measured dose volume points. The RA plans exhibited significantly greater low- and intermediate-dose exposure to the normal brain compared to all other techniques, especially at lower doses such as 8 and 5 Gy. For high-dose exposure, like V23 Gy and V18 Gy, the DCA and Hybrid plans were higher than the RA and HA plans, due to the lower CI. Regarding the maximum dose to other OARs, such as the brainstem, chiasm, and optic nerves, no clear trends were observed due to the small cohort size (Figures 3f-3i). Most differences were statistically insignificant (*P *> 0.05) based on the Wilcoxon signed-rank test. All dosimetric parameters are summarized in Tables 2-3.

**Table 2:** Median and interquartile range (IQR) of the CI, PCI, and GI for the different treatment techniques. CI, conformity index; PCI, Paddick conformity index; GI, gradient index; PTV, planning target volume; VMAT, volumetric modulated arc therapy

	Total PTVs						Only VMAT-applied PTVs					
	CI		PCI		GI		CI		PCI		GI	
	Median	IQR	Median	IQR	Median	IQR	Median	IQR	Median	IQR	Median	IQR
DCA	1.41	1.21-1.76	0.68	0.56-0.78	2.70	2.00-3.63	1.21	1.15-1.33	0.77	0.69-0.83	2.10	1.69-2.78
RA	1.11	1.02-1.27	0.85	0.75-0.91	3.39	2.86-4.64	1.01	0.95-1.04	0.93	0.91-0.94	2.71	2.18-3.22
HA	1.03	0.98-1.09	0.90	0.86-0.93	3.65	2.95-5.41	0.98	0.96-1.04	0.91	0.90-0.93	2.75	2.17-3.20
Hybrid	1.40	1.22-1.69	0.71	0.57-0.78	2.79	2.10-3.59	1.07	1.03-1.12	0.89	0.82-0.90	2.46	1.95-2.89

**Table 3:** Median and interquartile range (IQR) of the normal brain (cc) receiving 23, 18, 12, 8, and 5 Gy for the different treatment techniques. DCA, dynamic conformal arc; RA, RapidArc; HA, HyperArc

	V23 Gy		V18 Gy		V12 Gy		V8 Gy		V5 Gy	
	Median	IQR	Median	IQR	Median	IQR	Median	IQR	Median	IQR
DCA	19.1	9.1-33.9	32.9	16.5-60.3	70.7	39.0-136.3	152.8	92.4-297.7	364.2	185.7-577.2
RA	12.1	9.7-21.3	26.7	17.3-41.5	67.2	31.2-126.5	194.1	60.5-379.4	489.6	163.8-690.0
HA	11.1	6.0-19.3	20.6	11.7-37.3	47.0	28.2-86.9	97.6	55.9-183.7	270.8	113.8-522.9
Hybrid	17.3	9.7-26.1	30.0	17.7-45.7	66.8	39.1-106.9	138.1	83.3-253.9	332.9	179.7-512.5

**Figure 4:**
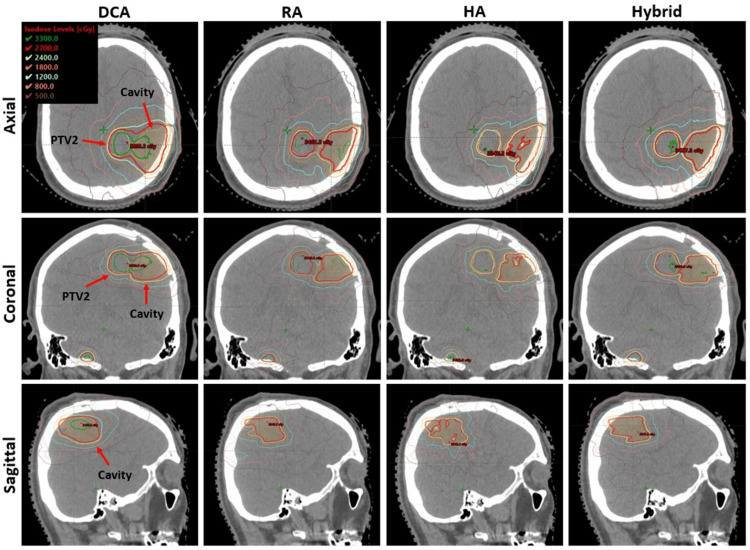
Dose distribution comparison for an example case (Case #2) with five targets, including an irregularly shaped cavity located close to another target. The Rx was 27 Gy in three fractions. Yellow and orange contours indicate PTV2 and the cavity, respectively, and the red isodose line indicates Rx. As shown, Hybrid improved dose conformity to the VMAT-applied target compared with DCA. VMAT, volumetric modulated arc therapy; DCA, dynamic conformal arc; RA, RapidArc; HA, HyperArc

**Figure 5:**
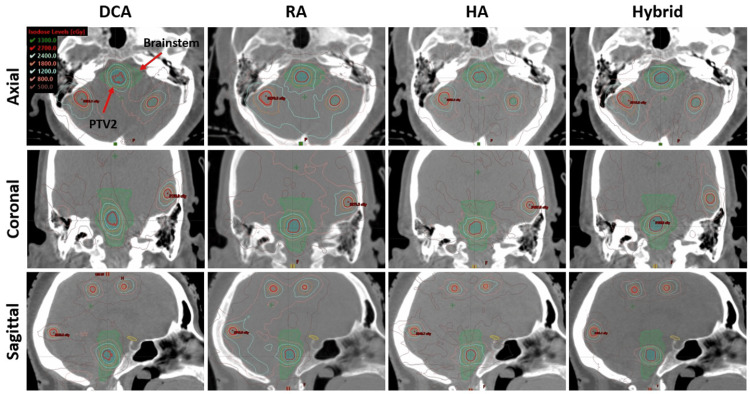
Dose distribution comparison for another example case (Case #6) with a total of 13 targets, including one target (PTV2) located in the brainstem. The Rx was 27 Gy in 3 fractions. Blue and green contours represent PTV2 and the brainstem, respectively, while the red isodose line indicates Rx. As shown, the Hybrid approach improved dose conformity and gradient to the VMAT-applied target compared with DCA. VMAT, volumetric modulated arc therapy; DCA, dynamic conformal arc; RA, RapidArc; HA, HyperArc

As a visual comparison of the dosimetric results, Figure 4 presents an example dose distribution for case #2, which has five targets, including a cavity in the left parietal lobe, with a prescription of 27 Gy in 3 fractions. The axial, coronal, and sagittal views highlight the cavity with PTV2, which is close to each other. Overall, the HA plan appears to have superior high-dose target coverage, better conformity, and lower low-dose spread. The hybrid plan shows significantly improved conformity compared to DCA, as seen in axial and coronal views, and it was comparable to that of RA and HA plans. Figure 5 shows another example case, which has a total of 13 targets and PTV2 located in the brainstem. The prescription dose is 27 Gy, and the maximum dose constraint for the brainstem was satisfied in all plans. As shown in Figure 5, all targets, including PTV2, were small and approximately spherical; however, the PCI for PTV2, located in the brainstem, was lowest with the DCA plan (0.77) due to the inherent limitations of the forward-planning approach. This was improved to 0.90 with the Hybrid plan, as shown in the right-hand column of the figure.

Finally, we compared the total beam-on times listed in Table 4, which were estimated based on the total monitor units divided by a 1400 MU/min output rate. On average, RA plans had the shortest beam-on time, followed by DCA, HA, and Hybrid plans. Planning time was shortest for DCA planning due to its automated workflow and longest for RA planning, which involved manual VMAT optimization. Hybrid planning took slightly longer than DCA planning but considerably less time than manual VMAT optimization.

**Table 4:** Comparison of beam-on time (minutes per patient) for delivery with a dose rate of 1400 MU/min. DCA, dynamic conformal arc; RA, RapidArc; HA, HyperArc

Patient#	# of target	DCA	RA	HA	Hybrid
1	3	2.38	2.53	2.28	3.85
2	5	3.11	3.00	3.63	4.60
3	2	2.04	2.15	2.69	2.50
4	6	3.47	1.97	3.24	3.60
5	6	3.44	3.10	3.79	4.53
6	13	4.62	4.33	5.62	5.33
7	4	2.32	1.80	1.94	4.23
8	13	5.13	3.91	4.90	5.37
9	13	5.26	3.48	4.32	5.33
10	8	3.75	2.76	3.50	4.58
Average ± std		3.55 ± 1.09	2.90 ± 0.79	3.59 ± 1.08	4.39 ± 0.86

## Discussion

In this study, we conducted the first clinical assessment of the BrainLAB Elements Hybrid Planning Technique, comparing it with traditional DCA and VMAT-based methods (RA and HA) for multi-target stereotactic radiosurgery. Our findings highlight the potential of Hybrid planning to overcome limitations of DCA while avoiding some disadvantages of highly modulated VMAT approaches.

Consistent with the previous report [11], the plans with VMAT-based techniques showed better conformity metrics (CI and PCI) compared to DCA plans. However, DCA plans often resulted in inadequate conformity or under-coverage for lesions with large and irregular shapes or in/near critical OARs. As illustrated in Figure 2, Hybrid plans achieved enhanced conformity beyond DCA plans and matched VMAT's performance for the VMAT-applied challenging target. As expected, DCA plans provided the lowest GI due to minimal modulation, while RA/HA plans resulted in higher GI. Importantly, Hybrid plans maintained DCA-like GI values (Table 2) while achieving better conformity, creating a clinically beneficial trade-off. These results indicate that Hybrid planning can provide a practical compromise between the simplicity of DCA and the flexibility of VMAT.

Normal brain dose analysis (Figure 3) showed HA plans minimized V23-V5 Gy, consistent with its automated HA-specific optimization algorithm, which automatically adjusts collimator angles and MLC modulations to minimize the dose spillage and interplay effects. On the other hand, RA plans increased low- and intermediate-dose brain exposure, which may be affected by the larger MLC and jaw openings compared with DCA, which increases the dose to the surrounding tissues, especially for intermediate-low doses. Hybrid plans preserved intermediate-to-low dose spillage similar to DCA plans, which is advantageous for reducing the risk of radionecrosis [15,16].

Efficiency continues to be a key factor in clinical adoption. Our data (Table 4) show that Hybrid plans have the longest average beam-on time, representing a *capacity tax* of approximately 22%-24% compared with other methods. However, from a Time-Driven Activity-Based Costing (TDABC) perspective, this machine-related cost is balanced by substantial professional labor savings. The automated, template-based workflow in Elements reduces manual intervention in planning and optimization, which minimizes inter-planner variability and standardizes the clinical process.

Our findings align with previous reports [11]. In our earlier study of single-isocenter, multi-target SRS with over 20 lesions, RA and HA showed better conformity compared to DCA, although RA specifically was linked to wider low-dose spread. This analysis builds on those findings by demonstrating that Hybrid planning can reduce DCA’s conformity limitations while mostly maintaining its favorable gradient profile. Vergalasova et al also noted that RA improved conformity but increased normal brain exposure at intermediate and low doses (e.g., V12 Gy) compared to conformal arc techniques [10]. Additionally, HA decreased the low-dose spread relative to RA, supporting our observation that HA resulted in the lowest normal brain exposure. Overall, these results suggest that Hybrid planning offers a middle ground, improving conformity without the significant low-dose exposure associated with VMAT.

The limitations of Hybrid planning became clear in two specific cases. First, when multiple targets were large and irregularly shaped at the same time, several lesions could be strong candidates for VMAT arcs. Because Hybrid only includes VMAT arcs at one target, it might not fully optimize conformity across all these targets, and a full VMAT approach could be better. Second, Hybrid might be less effective when small lesions are directly beneath larger targets along the beam paths that receive VMAT arcs. In these cases, the overlap of modulation can cause too much dose variation, and a purely VMAT-based plan might provide better maximum dose control and overall plan quality.

Several limitations in this study should be acknowledged. First, this was a retrospective planning study with a relatively small cohort of 10 patients and 73 PTVs. While this sample size provides a robust basis for target conformity and gradient assessment, the lack of statistical significance in certain OAR comparisons may be a direct result of the limited cohort size, rendering these specific findings preliminary. Also, the case selection was based on the planner's discretion, which introduces a source of bias and limits reproducibility across institutions. Second, we focused on dosimetric and efficiency endpoints without including clinical outcome data. Prospective studies evaluating toxicity, radionecrosis rates, and local control are warranted. Third, the beam-on time comparison was estimated by dividing the total number of monitor units by a fixed 1400 MU/min dose rate, which does not fully account for total treatment time, such as couch rotation time, gantry speed/dose rate variations, etc. Finally, as Hybrid planning is a newly commercialized feature, further investigations into workflow integration, QA procedures, and robustness across institutions will be essential.

## Conclusions

The BrainLAB Elements Hybrid Planning Technique provides a clinically valuable balance between DCA and VMAT for brain SRS that includes at least one challenging lesion. While this technical dosimetric evaluation demonstrates clear benefits in target conformity and dose gradients, future prospective studies are required to evaluate clinical outcomes, such as toxicity and local control.
